# Improve short-term survival in postcardiotomy cardiogenic shock by simultaneous use of intra-aortic balloon pumping with veno-arterial extracorporeal membrane oxygenation: Beware of confounders!

**DOI:** 10.1186/s13613-019-0550-7

**Published:** 2019-07-01

**Authors:** Patrick M. Honore, David De Bels, Sebastien Redant, Kianoush Kashani

**Affiliations:** 1 ICU Department, Centre Hospitalier Universitaire Brugmann-Brugmann University Hospital, Place Van Gehuchtenplein, 4, 1020 Brussels, Belgium; 2 Division of Nephrology and Hypertension, Division of Pulmonary and Critical Care Medicine, Department of Medicine, Mayo Clinic, Rochester, USA

We enthusiastically read the recently published retrospective study by Chen et al. [1] who demonstrated that simultaneous use of intra-aortic balloon pumping (IABP) together with veno-arterial extracorporeal membrane oxygenation (VA-ECMO) in postcardiotomy cardiogenic shock (PCS) patients improved short-term survival and reduced peripheral perfusion complications. In this study, 42 (28%) patients were on concomitant IABP and VA-ECMO [1]. While the study adds substantial value to the current knowledge, the current literature about the concomitant use of VA-ECMO and IABP remains controversial [2]. A more mechanical and pragmatic approach would be to state that VA-ECMO increases left ventricular (LV) afterload and decreases the blood flow in the coronary arteries due to retrograde blood flow, which can potentially deteriorate cardiac function while IABP could reduce these effects. Reduced LV afterload and increased blood flow in the coronary arteries by IABP theoretically promote myocardial recovery and could potentially improve survival (although improved survival was never shown) [2, 3]. In the baseline characteristics of patients before VA-ECMO implantation among the non-survivors, they were significantly more hypertension (35 vs. 15%; *P *< .004), secondary thoracotomies (39 vs. 19%; *P *< .007), cardiac arrests (34 vs. 11%; *P *< .001), bedside implantations 42 vs. 11%; *P *< .0001) and significantly less concomitant insertions of VA-ECMO and IABP (22 vs. 41%; *P *< .025) when compared with the study survivors [1]. All mentioned variables are well-described risk factors for increased mortality [3]. It is also reported that brain and kidney blood flow improves with concurrent initiation of IABP with ECMO [1]. Therefore, the question would be to find the mechanism by which concurrent initiation could reduce the need for continuous renal replacement therapy (CRRT) and decrease neurological complications [1]. Strategies aiming to prevent acute kidney injury (AKI) by increasing global blood flow to the kidneys have failed [4] as increasing blood flow mostly impacts the cortex while medulla remains hypoperfused. Therefore, it remains unclear why the use of IABP added to VA-ECMO in order to improve renal blood flow could significantly reduce the need for CRRT [1, 3, 4]. In order to decrease the chances of bias in the reported findings, the traditional AKI risk factors like diabetes mellitus, contrast exposure, the presence of shock and need for inotropes should be included in the comparison of these groups (VA-ECMO alone vs. VA-ECMO plus IABP) [1]. Adding IABP to VA-ECMO was not reported to increase limb ischemia [1]. This is in contradiction with a recent study by Yang et al. [5] which stated major vascular complications (MVCs) to be common and associated with higher in-hospital mortality among adult PCS patients receiving peripheral VA-ECMO support. Previously, obesity, concomitant IABP/ECMO, SOFA score at 24 h post-ECMO, and bleeding disorders were reported as independent risk factors for MVCs [5]. In conclusion and according to our interpretation, this very interesting study does not definitively show that adding IABP is improving short-term survival as many confounders could explain the observed difference in mortality.

## Data Availability

Not applicable.

## Abbreviations

PCS: postcardiotomy cardiogenic shock
IABP: intra-aortic balloon pumping
VA-ECMO: veno-arterial extracorporeal membrane oxygenation
LV: left ventricular
CRRT: continuous renal replacement therapy
AKI: acute kidney injury
MVCs: major vascular complications
